# Microwave Imaging and Emerging Applications

**DOI:** 10.1155/2012/252093

**Published:** 2012-05-09

**Authors:** Paul M. Meaney

**Affiliations:** Thayer School of Engineering, Dartmouth College, Hanover, NH 03755, USA

Active microwave imaging for biomedical has been proposed, studied, and implemented to varying degrees by a plethora of worldwide institutions for the last 30 years. Much of the interest has been based on the fact that tissue dielectric properties embody important information about physiological state and function. Early efforts concentrated on techniques involving classic techniques such as diffraction imaging and the Born and Rytov approximations which ultimately proved too limited for the high degree of field scattering involved with electromagnetic fields. At a time of rapidly increasing computational power and speed, the electromagnetics field quickly adopted these capabilities for developing improved forward and inverse modeling tools. This was especially convenient in that hardware implementations could be rapidly simulated and validated without the considerable time and expense of fabrication and testing. The evolution of numerical capabilities has been considerable to the point where fully 3D software packages, both customized and commercial, can represent complex geometries in reasonable time frames with exquisite resolution. 

Microwave imaging will always be a hybrid between numerical modeling and hardware. The two principle approaches today involve some form of tomography or 3D inversion and radar-based techniques. The former primarily utilizes transmission data, in combination with an inverse algorithm, produces maps of the interrogated dielectric properties. The algorithms fall under the category of classical parameter estimation problems and stem from a basic mathematical statement to minimize the differences between the measured and computed fields. A wide range of optimization problems have been implemented including, but not limited to, Gauss-Newton iterative schemes, genetic algorithms and backprojection techniques. These have often been 2D and 3D implementations and involved single and multifrequency modes. While this discipline has been dominated by simulation experiments, several efforts have expanded to at least the level of phantom validation investigations and even more advanced clinical implementations for breast cancer imaging. As with any technology, these realizations have involved important design trade-offs with respect to signal attenuation and detection strength, operating frequency, resolution, antenna selection, mechanical versus electrical array scanning, and many others. Early results have been encouraging and in an important development it is clear that the technique can recover accurate property maps that, as earlier hypothesized, can discern objects at levels well below the *λ*/2, Rayleigh criteria.

Radar approaches have also progressed substantially over the last decade or two. While much of the earlier work remained in the simulation realm, more recent efforts have included credible translation to experimental and even clinical implementations. The different approaches have involved either forward or backscattered measurement data and have explored air-coupled, liquid-coupled and contacting antenna techniques. This concept has been applied initially for the detection of high-contrast targets such as breast cancer with more recent efforts exploring approaches to more broadly characterize abnormalities. The classical challenges in this case involve designing broadband antennas, beam coverage of the entire target and generating sufficient measurement signal strength. More specific issues relating to breast cancer imaging involve the high degree of scattering at the skin. Early clinical efforts have produced refined and interesting breast images, especially for lower density breasts.

The application of microwave imaging to the breast cancer detection problem has singularly motivated the wide-ranging worldwide efforts in this technology. Breast cancer is an important worldwide health problem afflicting primarily woman. Treatment outcomes have been shown to be progressively more positive when the cancers are detected earlier. While conventional technologies such as X-ray mammography and MR do a good job of detecting and characterizing tumors, their effectiveness is particularly limited for more dense breasts. This has spurred the drive for investigating alternative approaches. As alluded to before, tissue dielectric properties can span a wide range of permittivity and conductivity values from very low values for fattier tissue to much higher values for high-water content tissues and a range of intermediate values depending on the composition. Initially the excitement for microwave breast imaging was based on the assumption that there was a very high property contrast between tumors and normal breast tissue and that these property differences would enable detection on a sub-centimeter scale. More recent studies have shown that the contrast is not necessarily as high and that the property variations are much more complex depending on the internal fibroglandular structure. However, this more nuanced understanding of the tissue properties provides real opportunities in the breast imaging area as the technology expands to roles in both breast cancer diagnosis and therapy monitoring. In fact, this opens up the opportunity for relating this recent data back to earlier, extensive studies by Ken Foster into the relationships between free and bound water with respect to tissue properties. These complex mechanisms have been hypothesized as prognostic indicators for cancer and may pave the way for dielectric properties becoming important biological markers.

As mentioned earlier, the earliest efforts in this arena were dominated by numerical modeling efforts. However, there is a realization within the community that for this technology to continue to be relevant, there needs to be concerted efforts to translate the various concepts into the clinic. This special issue is a step in that direction and demonstrates a maturing of the field. While there are some simulation studies within this collection, a good number of them deal directly with implementation challenges. Several in the radar area deal directly with acquiring in vivo measurements of a human breast. Another from the tomography techniques assesses the impact of surface waves to multipath contributions which can be an especially daunting problem in any radar, imaging, or communication system. These are healthy signs that the group understands that for it to continue to be relevant, it needs to steadily push these ideas into the clinic for real world validation. Simulations will always play an important role in this area, but the synergism with hardware implementations must be more heavily emphasized.

Beyond these classical motivations, this issue also highlights important points for the field as it moves forward. For instance, there is always room to explore new imaging techniques such as holographic approaches which may be particularly well suiting for the breast imaging situation. There are also clearly ways to improve on the overall approaches, especially when these methods can be combined with existing technologies such as proposed by the paper dealing with utilizing mammography in conjunction with radar approaches. Finally, while breast cancer imaging has dominated the landscape of microwave imaging for over a decade, we should not be blinded by other important health and commercial opportunities. The studies on bone dielectric property variations with respect to mineral density set the stage for applying microwave imaging in the area of osteoporosis detection and bone health monitoring which is becoming a major health issue with our rapidly aging population. This is only one of a range of important health issues where microwave imaging could make a substantial impact.

*Paul M. Meaney* *Paul M. Meaney*

